# Ventricular Tachycardia Storm - An Atypical Presentation of  Sarcoidosis Exacerbation

**Published:** 2009-11-01

**Authors:** Subba Reddy Vanga, James Vacek, Loren Berenbom, Dhanunjaya R Lakkireddy

**Affiliations:** Mid-America Cardiology, Kansas University Hospital, Kansas City KS

**Keywords:** Cardiac Sarcoidosis, VT Storm

## Abstract

Management of ventricular tachycardia (VT) storm in a patient with an implantable cardioverter-defibrillator (ICD) is a challenging medical emergency. We describe a patient with cardiac sarcoidosis (CS) and an ICD who is admitted with VT storm. Management of VT was difficult due to resistance to multiple antiarrhythmic drugs. He responded to immunosuppressive therapy supporting active CS as the cause of his VT. This case suggests that CS may underlie some cases of refractory VT and that immunosuppressive therapy may be effective in controlling this arrhythmia.

## Introduction

Sarcoidosis is a disease of unknown etiology and protean manifestations. Cardiac involvement is not uncommon. Rhythm disturbances are an important manifestation and can lead to sudden cardiac death. We report a patient with CS who presented with ventricular tachycardia storm resistant to traditional antiarrhythmics. He responded well to corticosteroids and methotrexate.

## Case Report

**Figure 1:**
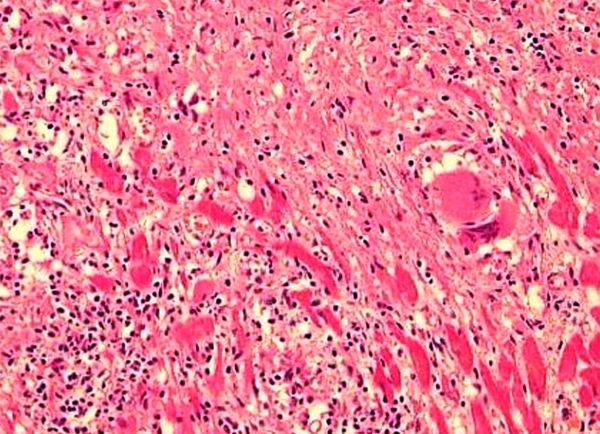
Myocardial biopsy of the patient showing non-caseating granulomas, typical of sarcoidosis

A 46-year-old male with hypertension, type 2 diabetes mellitus and obstructive sleep apnea was diagnosed histologically with pulmonary sarcoidosis 5 years previously. He was treated with oral prednisone tapered over 6 months. Three years later, he developed Mobitz 1 second-degree atrioventricular block. Six months after that he developed congestive heart failure (CHF) with left ventricular ejection fraction (LVEF) of 40%. A coronary angiogram did not reveal any significant stenosis. Cardiac MRI showed concentric left ventricular hypertrophy with hyperenhancement. Endomyocardial biopsy confirmed extensive myocardial sarcoidosis. (Figure 1). He had progressive decline of his LVEF to 20% and developed left bundle branch block (QRS 165 msec). He was treated with oral prednisone 60mg daily. In light of functional class III CHF despite optimal medical therapy, a cardiac re-synchronization ICD (Medtronic Concerto CI54DWK) was implanted. Over the next two months, he received six shocks for sustained VT and was placed on sotalol 120 mg twice daily with continued oral steroid therapy. His CHF symptoms improved and arrhythmia was under control.

**Figure 2:**
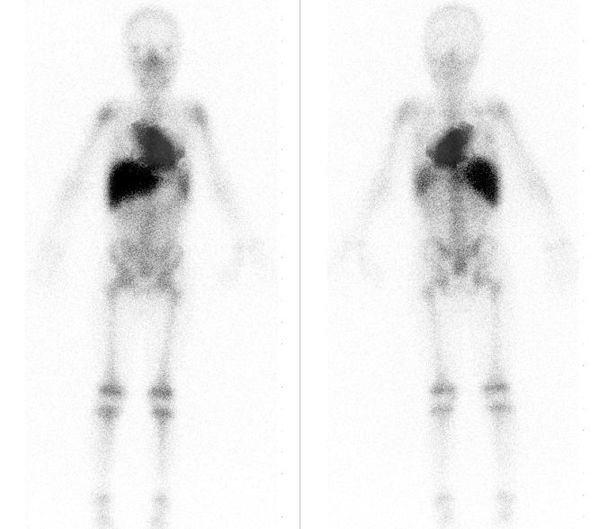
Gallium-67 whole-body scan of the patient showing intense uptake in heart and mediastinum

Five months after CRT-D implantation, he was admitted with multiple episodes of VT despite being on oral prednisone 30 mg daily and sotalol. He was treated successfully with antitachycardia pacing 56 times, and he was cardioverted 12 times over the course of 24 hours. VT was right bundle inferior axis with negative precordial concordance suggesting an apical origin. All episodes of VT were of similar morphology with a cycle length of 300 mSec. Lidocaine, mexiletine and amiodarone reduced the frequency of sustained VT episodes, but he continued to have episodes of symptomatic non-sustained VT. LVEF was 15-20%. A whole body gallium scan demonstrated uptake in the heart consistent with exacerbation of cardiac sarcoidosis (Figure 2). High dose intravenous steroid therapy (methylprednisolone 60 mg every 6 hours) was initiated in place of chronic oral steroid therapy and VT resolved within 12 hours. Oral methotrexate was added as a steroid sparing strategy. Sotalol was continued and other antiarrhythmics were stopped. Three days later, he was discharged on methotrexate (15mg/week PO) and a tapering dose of oral prednisone. At 3 and 6 months follow-up visits, his CHF was compensated with an LVEF of 25-30% and he was free of ventricular tachycardia on device interrogation. Repeat gallium scan at 6 months showed no significant uptake in the heart.

## Discussion

Management of arrhythmias in CS is difficult and effective control of VT often is not achievable by a single method of therapy. Acute inflammation with new granuloma formation that can disrupt myocardial fibers together with the patchy scarring associated with old healed granulomas can provide a substrate for new VT reentrant circuits. Antiarrhythmic drugs can facilitate occurrence of VT, as well as aggravate conduction abnormalities. Hypercalcemia, inherent to sarcoidosis; hypokalemia from concurrent corticosteroid use, as well as the interaction between acute phase reactants and antiarrhythmics can further complicate drug therapy [1].

Optimal therapy of ventricular arrhythmia associated with CS is not well established. Various regimens including corticosteroids, antiarrhythmic medications, ICDs and catheter ablations have been attempted. Steroids improved overall mortality in CS [2]. Steroids are believed to be capable of attenuating the inflammatory response and slowing subsequent fibrosis. Although corticosteroids help in suppressing arrhythmia secondary to sarcoid exacerbation, they may be of little use in the management of VT from scarred myocardium. This can explain the recurrence of VT despite treatment with corticosteroids in previously reported cases [3-4]. Active sarcoid exacerbation may cause VT in patients who are already on treatment with moderate to high doses of corticosteroids as seen in this patient. Immunosuppressive therapies are recommended as a steroid sparing strategy in the management of sarcoidosis and can be beneficial in controlling arrhythmia.

A stepwise approach in the management this malignant arrhythmia was studied [5]. ICD implantation, immunosuppression with corticosteroids, then up to two antiarrhythmic drugs were used (in that order) before patients were taken to the EP lab for mapping and ablation. 9 of the 21 patients with sustained VT underwent ablation. 44 VTs were induced and 31 were ablated. VT burden was reduced more than 98% in the first 3 months post ablation. The mechanism of VT in virtually all patients was re-entry [5,6].

Cardiac Resynchronization Therapy (CRT), which is an adjunct treatment to pharmacological therapy in patients with advanced heart failure, may have potential proarrhythmic effects [7]. Although rare, CRT has been shown to cause VT [8] and electrical storm [9]. Most CRT associated ventricular proarrhythmia was seen in ischemic cardiomyopathy patients soon after biventricular pacing lead implantation, and improved with cessation of biventricular pacing [8,9]. This patient tolerated CRT well for months. The presence of extensive myocardial sarcoidosis and lack of recurrent VT during the follow-up period despite continued CRT makes CRT related proarrhythmia an unlikely mechanism of VT in our patient. Electrophysiology study with ablation can be undertaken in CS, but VT circuits can change as inflammation waxes and wanes. VT may be ablated only to reoccur as the substrate shifts. Ablation should be considered as an option in those patients with recurrent VT that are unresponsive to antiarrhythmic and immunosuppressive drugs.

## Conclusion

Cardiac sarcoidosis related arrhythmia can be very difficult to manage. Because of increased awareness of the risk of sudden cardiac death and early ICD implantation, CS patients can survive to present with VT storm, which is not uncommonly resistant to standard antiarrhythmic drugs. While corticosteroids are mainstay of therapy to control the acute inflammation and hence related arrhythmia, immunosuppressive therapy such as methotrexate should be considered early if steroid resistance develops and before proceeding to catheter ablation.
